# The Influence of Action Effects in Task-Switching

**DOI:** 10.3389/fpsyg.2012.00595

**Published:** 2013-01-07

**Authors:** Sarah Lukas, Andrea M. Philipp, Iring Koch

**Affiliations:** ^1^Institute of Psychology, Rheinisch-Westfälische Technische Hochschule Aachen, Aachen UniversityAachen, Germany; ^2^Institute of Psychology and Education, Ulm UniversityUlm, Germany

**Keywords:** cued task-switching, action control, task set, task selection, preparation time

## Abstract

According to ideomotor theories, intended effects caused by a certain action are anticipated before action execution. In the present study, we examined the question of whether action effects play a role in cued task-switching. In our study, the participants practiced task-response-effect mappings in an acquisition phase, in which action effects occur after a response in a certain task context. In the ensuing transfer phase, the previously practiced mappings were changed in a random, unpredictable task-response-effect mapping. When changed into unpredictable action-effects, RT as well as switch-costs increased, but this occurred mainly in trials with short preparation time and not with long preparation time. Moreover, switch costs were generally smaller with predictable action-effects than with unpredictable action-effects. This suggests that anticipated task-specific action effects help to activate the relevant task-set before task execution when the task is not yet already prepared based on the cue.

## Introduction

The question of how human actions are mentally controlled is one that has been investigated since the early beginnings of cognitive psychological research in the nineteenth century (for a historical review see Stock and Stock, 2004; Shin et al., 2010, for a more recent review). According to the ideomotor principle, actions are controlled by internal processes – the anticipation of the intended action-effect (James, 1890). That is, the intended result of the action is already mentally represented before the action execution and controls the action.

A challenge for action control is to select and execute the appropriate action among a variety of feasible actions in a certain situation and to ignore or inhibit possibly competing actions. This implies that the system of action-selection processes has also to be very flexible. The context in which an action is performed is highly important (Brass et al., 2003; Kunde et al., 2007; Braverman and Meiran, 2010) and can change easily. This context and all other important components that are necessary to perform an action (or a task) are assumed to be mentally represented in a *task set* (see, e.g., Prinz et al., 2009). A task set contains information for example about the appropriate class of task-relevant stimuli, the stimulus-dimension, and the response mode (e.g., Philipp and Koch, 2010). The nature of a task set and its flexibility has been investigated in a variety of studies using the task-switching paradigm (see, e.g., Monsell, 2003; Kiesel et al., 2010; Koch et al., 2010; Meiran, 2010, for reviews).

In a typical task-switching experiment, subjects are instructed to switch between two reaction tasks that appear in a random order. It is usually found that performance [reaction time (RT) and error rate] is worse after a task switch than after a task repetition. These *switch costs* are supposed to reflect, at least partly, “executive” processes that are needed for switching between tasks. These executive processes imply on the one hand the maintenance of a mental representation of the current task. On the other hand, flexibility is required and the ability to inhibit the just executed task set. This includes the ability to overcome the persisting task activation (which is called *task-set inertia*), to shift attention to the new, currently relevant task-set, and to activate the new task-set (e.g., Allport et al., 1994; Rogers and Monsell, 1995; Meiran, 1996; Kiesel et al., 2010).

Action effects, that is, effects that happen as a consequence of the specific task execution, might help to reduce the conflict of ambiguous task-sets. When referring to action effects, we are referring to effects or events that take place after the response, as explicit consequence of the response. Examples for action effects are the tone that is heard after pressing a piano’s keyboard or the light that is turned on after pressing the light switch.

The influence of action effects that occur after response execution can be realized by the *anticipation* of the effect, which happens temporally before response execution, as assumed in the ideomotor principle. For example, when playing a piano, the pianist anticipates already the tone he/she wants to produce before pressing the key (see for empirical evidence e.g., Keller and Koch, 2008). That this kind of anticipation not only takes place in humans but also in animals was shown in so-called “differential outcome” studies (for a review, see Urcuioli, 2005). In these studies, animals learn different responses to different stimuli that lead to different outcomes. It is assumed that in learning of stimulus-response-outcome contingencies, the outcomes (or action effects in our terminology) are part of what is learned and are not merely “reinforcers.” That is, they serve as anticipatory cue to guide behavior by adding up to discrimination of the possible action alternatives (see Urcuioli, 2005).

When we assume that action effects influence response selection, action effects might also play a crucial role in task-switching. However, as far as we know, the impact of action effects have been neglected in task-switching research, even though in the literature, the concept of task-set appears often in conjunction with action control (e.g., Kunde et al., 2007; Hommel, 2009; Prinz et al., 2009). However, only few studies indeed combined a task-switching paradigm with effects that took place after the response.

Kiesel and Hoffmann (2004) reported one of the few studies that added action effects to a task-switching paradigm. They used this paradigm to create two different contexts (horizontal vs. vertical arrangement of a target) in which the same action (a key stroke) was performed, leading to two different action-effects (short/fast vs. long/slow movements of the target in the horizontal and vertical arrangement, respectively). Reactions were slower in the slow-movement context and faster in the fast-movement context, although these target movements occurred *after* the response. Hence, it was shown that action-effect associations are acquired according to the context and that the basically same actions are influenced by different (context-dependent) effect anticipation.

In another study, Ruge et al. (2010) provided task-related action effects in one condition and task-unspecific feedback about the correct execution of the task in another condition. Two target stimuli were horizontally *and* vertically aligned. A cue indicated if the position of the horizontal or the vertical target should be determined. The task-related action effect was a red light-up of the target in the position which indicated the correct response. This means, if the correct response was “right,” the target on the right side appeared red as consequence of the response, whereas the vertical target stayed colorless; and if the correct response was “up,” the target turned red on the up-position, whereas the horizontal target stayed colorless. The action effects were thus semantically associated with the correct response. The task-unspecific effect was just a feedback with the information whether the task was executed correctly or not. The authors found a significant two-way interaction of task transition and type of effect for trials with a long cue-target interval (CTI; i.e., 1500 ms). In the task-specific effect condition, residual switch costs were reduced compared to the unspecific effect condition. That is, anticipating task-specific action effects might help to discriminate the task sets of the upcoming trial and select the appropriate one. However, in this study, only trials with a long CTI were analyzed so that no statement can be made about how action effects influence task performance in trials with a short CTI.

Moreover, the question of whether the anticipation of action effects indeed influences the task activation process was not in the focus of these studies. According to ideomotor theories, action effects are mentally represented *before* response execution (e.g., James, 1890; Greenwald, 1970). That is, if action effects are mentally represented before response execution, the representation of action effects should influence the activation of a task set, for example by helping to discriminate the task sets.

The aim of the present study was to examine the role of action effects in task-switching. To this end, we devised a novel transfer paradigm. In order that intended effects can trigger actions, the regularities between the action and the following effect have to be acquired, so that stable action-effect associations result in effect anticipation prior to action execution (e.g., Elsner and Hommel, 2001, 2004; Dutzi and Hommel, 2009; cf. also Zießler et al., 2004). For this reason, we divided our experimental paradigm in two phases. In the first phase, the participants had to learn the task-response-effect contingencies so that task-response-effect associations could be established. These effects can then be anticipated after the cue is presented. In the second, transfer phase, the previously practiced mappings were changed into a random mapping, so that valid anticipation of action effects was no longer possible.

We assumed that in the acquisition phase the expected action-effects are anticipated after the presentation of the cue before task execution, probably during action planning (see Zießler et al., 2004), thus helping to further disambiguate the task set relative to the currently irrelevant, competing task set. We would like to note here that with the cue, both possible action-effects for the appropriate task (i.e., that occurring after the left or right response key-press) are anticipated. If the previously experienced task-response-effect mappings, however, are not valid anymore, as in the transfer phase, the implementation of the task-set should take more time because the effects as additional cues for task-set disambiguation cannot be utilized any longer prior to task activation. This prolongation, if observed, might be due to additional monitoring processes that “double-check” if the implemented task-set is appropriate.

If the influence of anticipated action-effects needs time to build up, we would assume an influence of task preparation time. To examine the influence of preparation time on the impact of action effects in task-switching, we manipulated the cue-target interval. If the task-response-effect association becomes stronger with more time to activate the task set, then we would expect a more pronounced increase in RT and switch costs for long preparation intervals compared to short preparation intervals. However, with the presentation of the cue, the cue-task association is as well retrieved, entailing a sustained bias to the relevant task components (Meiran, 2000; Koch and Allport, 2006; Meiran et al., 2008), so that the influence of anticipated action-effects becomes smaller when the task is already well prepared based on the cue. Given that the action effects are nominally task-irrelevant (and occur only *after* task execution), we assume that they play a stronger role primarily when the task is not yet fully prepared (i.e., with short CTI).

## Materials and Methods

### Participants

Twenty-four students of the RWTH Aachen university (19 female, 5 male; mean age = 23 years) took part in the experiment. They received partial course credit or 8€. The participants were equally and randomly assigned to the experimental vs. the control group. Informed consent was obtained from all subjects, and the experiment was performed according to the ethical standards of the Declaration of Helsinki.

### Apparatus, stimuli, and tasks

The experiment was programmed with the experimental runtime system ERTS (Version 3.33e, BeriSoft Cooperation, Frankfurt am Main, Germany). Participants sat in front of a screen with a viewing distance of approximately 60 cm. The stimuli consisted of digits ranging from one till nine, without the five. They appeared in white on a black background at the center of the screen with a height of 1.4 cm (vertical visual angle: 1.34°).

The two tasks were two numerical judgment tasks. In one task, the participants had to decide if a presented number was greater or less than five (i.e., the magnitude task). In the other task, the participants had to decide if the presented number was odd or even (i.e., the parity task). The tasks switched randomly and were indicated by a cue. Each number was framed by either a diamond or a rectangle. If the number was framed by a diamond, the magnitude task was required; if the number was framed by a rectangle, the parity task was required. The diamond was 3 cm high and 3 cm wide (vertical and horizontal visual angle: 2.86°). The rectangle was 3.6 cm high and 3.5 cm wide (vertical visual angle: 3.44°; horizontal visual angle: 3.34°). Responses were to be made by manually pressing one of two response keys (i.e., the left and right Alt-key) with the left or right index finger. Participants were instructed to press left for a smaller or an odd number and to press right for a greater or an even number. If the response given by the participants was correct, an action-effect occurred. The effects were assigned to the task and the response in the experimental group in the first eight “acquisition blocks.” For example, for the magnitude task, visual action-effects and for the parity task, auditory action-effects occurred. If the response was “less” (left key-press), the background of the screen turned yellow and if the response was “greater” (right key-press), the background of the screen turned green. For the parity task, a honking tone was presented after the correct response for “odd” (left key-press) and a ringing tone after the correct response for “even” was presented (right key-press). The mapping between task, response, and action-effect was counterbalanced across participants in the experimental group. For the control group, the action effects were completely random, that is, participants could not establish an association between task, response, and action-effect and thus could not anticipate the action effect. If the response was wrong, nothing happened, the background just turned black in both groups. Participants were instructed to react as fast and as correct as possible. They were informed in both groups that the effects happening after the key-press should be used as feedback whether the task was performed correctly.

### Procedure

Each trial started with a cue, which appeared in half of the trials 100 ms prior to the target stimulus (CTI of 100 ms) and in the other half of the trials 900 ms prior to the target stimulus. The CTI duration varied randomly. Cues and stimuli stayed on the screen until a response was given. For both CTI levels, an action effect was presented for 200 ms immediately after a correct response. That is, the response-cue interval (RCI) was held constant at 400 ms and the response-stimulus interval (RSI) was 500 ms for trials with a short CTI and 1300 ms for trials with a long CTI.

Before the experiment started, the participants performed a practice block with 16 trials. The experiment consisted of eight acquisition blocks and one transfer block. The acquisition blocks as well as the transfer block comprised 96 trials each plus four warm-up trials that were not recorded. Altogether, one session lasted about 45 min.

In the experimental group, the task-response-effect associations were predictable so that a mental task-response-effect association could be established by the participants. In the transfer block, the learned associations were changed into random action-effects as in the control group. There was no difference between the acquisition blocks and the transfer block in the control group. To analyze the influence of the change in the action-effect assignment, performance in the last acquisition block (Block 8) was compared with that in the transfer block (Block 9).

### Design

Task transition (switch vs. repetition), block (Block 8 vs. Block 9), and CTI (short vs. long) were used as independent within-participants variables; group (experimental vs. control) was used as independent between-participants variable. RT and error rate were measured as dependent variables. Significance was tested at α = 0.05.

## Results

In addition to the four non-registered warm-up trials, the first recorded trial of each block was excluded from analysis because it could not be categorized as a switch or repetition trial. All trials with RT less than 200 ms or exceeding 3 SD of each participant’s mean were discarded as RT outliers (i.e., 2%). Furthermore, all incorrect trials and that following an incorrect trial were not included in the analysis. Errors occurred in 4.8% of the trials.

### RT

**Figure 1:**
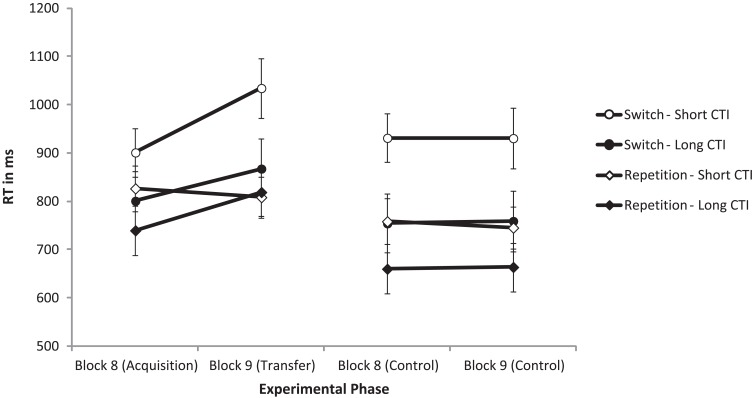
**Mean reaction times (RT) in ms as a function of task transition, experimental phase, group, and cue-target interval (CTI)**.

The data were submitted to a four-way mixed analysis of variance (ANOVA) with the independent variables task transition, block, CTI, and group. To forestall the most important result: the four-way interaction of task transition, block, CTI, and group was significant, *F*(1, 22) = 5.49, *p* = 0.029, η^2^ = 0.2 (see Figure 1). That is, the increase of switch costs in the transfer block with a short preparation interval takes place only for the experimental group, whereas there were no effects of block in the control group. To better understand this four-way interaction, below we split the analysis in the two experimental groups and analyzed them separately with two three-way ANOVAs. In the overall, four-way ANOVA, the main effect of group was not significant, *F* = 1.2. The only other interaction with the variable group was the two-way interaction of group and block, *F*(1, 22) = 4.98, *p* = 0.036, η^2^ = 0.185. Only in the experimental group, RT increased in Block 9 (from 817 to 882 ms), but not in the control group (776 vs. 775 ms). This interaction is also reflected in the split ANOVA, as described below.

For the *experimental group*, the three-way interaction of task transition, block, and CTI was significant, *F*(1, 11) = 12.34, *p* = 0.005, η^2^ = 0.529. Switch-costs increased significantly from Block 8 (75 ms) to Block 9 (226 ms) in short CTI trials, whereas they even somewhat decreased numerically from Block 8 (61 ms) to Block 9 (49 ms) in long CTI trials. Further, the two-way interaction of task transition and CTI was significant, *F*(1, 11) = 6.7, *p* = 0.025, η^2^ = 0.378, showing that switch-costs decreased from 150 ms for short CTIs to 56 ms for long CTIs. The main effects of task transition, *F*(1, 11) = 30.03, *p* < 0.001, η^2^ = 0.732, block, *F*(1, 11) = 10.3, *p* = 0.008, η^2^ = 0.484, and CTI, *F*(1, 11) = 11.05, *p* = 0.007, η^2^ = 0.501 were significant, too. RT was shorter for repetition trials (798 ms) than for switch trials (901 ms), for Block 8 (817 ms) than for Block 9 (882 ms) and for long CTIs (807 ms) than for short CTIs (893 ms). To go more into detail, and understand better the three-way interaction, we further split the analysis in two two-way ANOVAs for short and long CTI trials.

The trials with a short CTI showed a significant two-way interaction of task transition and block, *F*(1, 11) = 6.75, p = 0.025, η^2^ = 0.380, indicating that switch-costs increased in the transfer block. Further, the main effect of task transition was significant, *F*(1, 11) = 35.38, *p* < 0.001, η^2^ = 0.763 whereas the main effect of block was just not significant, *F*(1, 11) = 4.13, *p* = 0.067, η^2^ = 0.273. For the trials with a long CTI, the two-way interaction of task transition and block was not significant, *F* < 1. The main effect of task transition failed to reach significance, *F*(1, 11) = 4.07, *p* = 0.069, η^2^ = 0.270, but the main effect of block was significant, *F*(1, 11) = 11.26, *p* = 0.006, η^2^ = 0.506. RT was increased from Block 8 (770 ms) to Block 9 (843 ms).

In contrast to the experimental group, in the *control group*, the three-way interaction of task transition, block, and CTI was not significant, *F* < 1. The well-known two-way interaction of task transition and CTI was significant, *F*(1, 11) = 11.03, *p* = 0.007, η^2^ = 0.501. It reflects a reduction of switch costs with a long preparation interval (short CTI: 179 ms, long CTI: 95 ms). The main effects of task transition, *F*(1, 11) = 20.74, *p* = 0.001, η^2^ = 0.653, and CTI, *F* (1, 11) = 47.69, *p* < 0.001, η^2^ = 0.813 were significant. RTs were faster in repetition trials (707 ms) than in switch trials (844 ms) and faster with a long preparation interval (709 ms) than with a short preparation interval (841 ms). There was neither a main effect of block, nor an interaction including this variable (*F*s < 1).

In the analysis of trials with a long preparation interval in the experimental group, we found small and non-significant switch-costs, like in the study of Ruge et al. (2010). Hence, we further investigated if we could also find diminished residual switch-costs with predictable action-effects compared to unpredictable action-effects already in the acquisition phase. In order to do this, we analyzed RTs averaged across the first eight acquisition blocks (see Table 1). These data were submitted to a three-way ANOVA with a 2 (task transition) × 2 (CTI) × 2 (group) design. Noteworthy, the interaction of transition and group was significant, *F*(1, 22) = 20.02, *p* < 0.001, η^2^ = 0.476. Switch costs were higher in the control group (190 ms) than in the experimental group (50 ms). Also, the three-way interaction of transition, CTI, and group was significant, *F*(1, 22) = 4.51, *p* = 0.045, η^2^ = 0.17. Switch costs were especially high in the control group in short CTI trials (250 ms), but could be reduced with a long CTI (130 m), which is a reduction of 48%. However, in the experimental group, switch costs were even more reduced with a long CTI (from 75 to 24 ms), which is a reduction of 68%. In addition, also the expected interaction of transition and CTI was significant, *F*(1, 22) = 28.29, *p* < 0.001, η^2^ = 0.563. Switch costs were higher in trials with a short CTI (162 ms) than with in trials with a long CTI (77 ms). As expected, the main effect of transition, *F*(1, 22) = 58.41, *p* < 0.001, η^2^ = 0.726, and of CTI were significant, too, *F*(1, 22) = 105.26, *p* < 0.001, η^2^ = 0.827. Neither the main effect of group was significant, *F* = 1.3, nor the interaction of CTI and group, *F* = 2.29.

**Table 1:** **Mean RT in ms (and SE) of the first eight blocks (acquisition phase) as a function of task transition (repetition vs. switch), CTI (short vs. long), and group (Experimental group vs. Control group)**.

			Cue-target interval	
		Task transition	Short	Long
Group	Experimental group	Switch	989 (60)	855 (56)
		Repetition	913 (43)	831 (41)
	Control group	Switch	1017 (60)	811 (56)
		Repetition	767 (43)	681 (41)

To check at which point of time the switch-costs decreased for the experimental group, we additionally took a look at the switch costs in the first eight blocks for the experimental and the control group. The main effect of group was not significant, *F* = 1.2. Already in the first two blocks, the switch costs were smaller in the experimental group (Block 1: 81 ms; Block 2: 47 ms) than in the control group (Block 1: 267 ms; Block 2: 280 ms). The interaction of transition and group in the first two blocks was accordingly significant, *F*(1, 22) = 20.02, *p* < 0.001, η^2^ = 0.476.

### Errors

For the error rate, the four-way ANOVA for Block 8 and 9 was conducted. It revealed only a main effect of task transition with fewer errors in repetition trials than in switch trials (3.9 vs. 5.97%), *F*(1, 22) = 7.86, *p* = 0.01, η^2^ = 0.263. No other main effect or interaction was significant, *F*s < 1.9.

Also in the first eight blocks, no difference between group was shown, *F* < 1, nor an interaction with switch costs.

## Discussion

The aim of the present study was to investigate the role of action effects in task-switching. In order to examine this question, we designed a task-switching paradigm in which task-irrelevant, but predictable action-effects occurred. The task-response-effect mappings were practiced in eight acquisition blocks, so that action effects could be reliably anticipated. In a ninth (transfer) block, the action effects were random, so that the effects could no longer be anticipated in a task- and response-specific way. Additionally, the CTI was manipulated to examine effects of task preparation. The results showed that going from predictable (i.e., anticipated) to unpredictable action-effects increased both RT and switch costs. However, this influence of anticipated action-effects was found only in trials with a short CTI.

The fact that task-switching performance is impaired when previously learned action-effects cannot be anticipated anymore in a task-specific way relative to when action effects are unpredictable throughout the experiment shows that the predictable effects have been *anticipated* because otherwise an influence of stimuli that occur after response execution would be quite inexplicable. Hence, this influence of action effects is consistent with the already existing literature on effect anticipation (e.g., Elsner and Hommel, 2001; Koch and Kunde, 2002; Kunde et al., 2007). Moreover, the finding that the influence of action effects depends on both CTI and task transition (i.e., is largest on switch trials with short CTI) rules out a general “surprise” effect because this should be comparable across conditions. Note that even the concept of surprise presupposes that an expectation, that is, anticipation is violated, so that surprise actually assumes anticipation. However, we assume that the role of anticipated action-effects is more specific and lingering, because it cannot be ignored easily. A mere surprise effect should be transient and easier to overcome.

To account for the data, we assume that in short CTI trials, the task set of the previous task is still activated, so that a new implementation is not necessary in task repetition trials. But for switch trials, a new task-set has to be activated, which is a time-consuming process. In implementing the relevant task-set, also the task-response-effect associations are activated, which helps in further disambiguating the task set so that less interference between competing task-sets occurs. That is, for the magnitude task, the two visual action-effects and for the parity task, the two auditory effects are anticipated. The anticipation helps to activate and implement the correct task-set, reducing the switch costs. However, if the action effects are not predictable anymore, they cannot facilitate selection of task or response any longer, yielding higher RT in switch trials. In contrast, in trials with long CTIs, participants have enough time to activate the relevant task-set based on the cue, so that the switch-specific component of the facilitative influence of anticipated action-effects disappears. However, anticipated action-effects still show a general beneficial influence in task switches and repetitions alike, probably because the task-specific action-effect anticipation in the response selection process helps keeping the task sets better separate and thus counters stimulus-based task interference (e.g., Rogers and Monsell, 1995; Koch and Allport, 2006). Note that the idea that not the stimuli themselves are made more distinguishable from each other, but the task context or the required action is also endorsed by studies examining the differential outcome effect in animals (e.g., Honig et al., 1984; cf. also Urcuioli, 2005).

In explaining our data, we would like to point out again that the action effects are completely irrelevant to the task. Keep in mind that the predictable action-effects occur *after* the response and could therefore easily have been ignored. Nevertheless, they influenced task-switching performance, and specifically switch-costs, when the preparation time was short. The increased switch-costs as well as the increased RTs for long CTIs are an indication that the action effects could not be ignored, and thus affected task performance.

Action-selection can be externally controlled by stimuli or internally by goals and/or intentions. In earlier studies, it was shown that learning of task-effect associations only took place in an intention based experimental setting (Herwig et al., 2007; Herwig and Waszak, 2009). Thus, it is noteworthy that in our study, task-effect associations were built up even though learning was rather stimulus-based on the cue than intention based. However, Herwig and Waszak (2009) also stated that under certain experimental conditions, like more complex S–R mappings, action effects might become more important, thus allowing also “ideomotor” learning for stimulus-based actions. Moreover, it was recently shown that also stimulus-based settings can yield response-effect expectancies (Pfister et al., 2010) or effect-response preferences after a forced-choice acquisition phase (Pfister et al., 2011). Our study provides additional evidence that under forced-choice, stimulus-based action-effect learning takes place and influences the response behavior.

One would assume that the action-effect associations built up in the acquisition phase is weakened in the transfer phase. Consequently, the increase in switch costs should be higher in the beginning of the transfer block than at a later point of time. However, there is an alternative explanation: one could argue that participants realize that the action effects are not useful anymore to add up to activate a task set and adopt a task-processing strategy like in the control condition. As we have seen, switch costs are higher in the control group than in the experimental group. Consequently, an increase of switch costs at a later point of time in the transfer block should be assumed. We checked which of the two explanations holds by comparing the switch costs in the first half of the transfer block to the switch costs in the second half of the block for each group. However, we could not find an interaction of task transition, block half, and group. Switch costs changed neither in the control group nor in the experimental group significantly from the first to the second half of the last block. As already mentioned before, this finding speaks additionally against a mere “surprise effect” as surprise should have only a transient effect.

Our results also showed a difference in the switch costs between groups already in the acquisition phase. The experimental group with the predictable action-effects revealed smaller switch costs than the control group with the random action-effects, corroborating the results of the study of Ruge et al. (2010). However, the switch costs were mainly reduced because the repetition trials in the experimental group showed higher RT than the repetition trials in the control group. The RT in the control group and the resulting switch costs of about 190 ms are to be expected for a parity-magnitude task-switching experiment (cf. e.g., Arrington and Logan, 2005). That is, providing action-effects might lead to an aggravation of performance in repetition trials. It is possible, that the higher information content with predictable action-effects leads to longer task-processing time, during which the action effects are anticipated. But this is only observed in repetition trials because action-effect anticipation can be done simultaneously in the additional time in which a task switch is prepared. As soon as this additional information processing is done, the anticipated action-effects help to disambiguate different task-sets: In the experimental group, switch costs were not additionally increased in general, but they were proportionally more strongly reduced by a long preparation interval. In contrast to the action effects in the study of Kiesel and Hoffmann (2004) and Ruge et al. (2010), the action effects in our study were arbitrary and task-irrelevant. Nevertheless, they can easily be integrated in the task set. It can be assumed that, for example, visual action-effects, like the yellow or green background for the magnitude task, are incorporated as component to the task-set. This would imply that action effects within one task should be similar and easily distinguishable from the action effects of the alternative task, so that they have a positive effect in task performance (cf. Honig et al., 1984). However, action effects do not have to be similar to the respective response, concerning for example an ideomotor-compatible response-modality/effect-modality mapping (Greenwald, 1972), spatial compatibility (Ansorge, 2002; Pfister et al., 2010), numerical magnitude compatibility (Badets et al., 2012), verbal response-effect compatibility (Koch and Kunde, 2002), or the compatibility between key-alignment and respective tone production (Keller and Koch, 2008) in order to become associated with a task. This conclusion is also corroborated by studies of Hommel (2009), in which he investigated the influence of irrelevant action features. He found reliable correspondence effects for irrelevant action-effects. He concluded that not only intended, but also non-intended action effects are automatically integrated in the action code (*automatic integration hypothesis*). Although intended action-effects may be weighted more in the action code (Hommel, 2009; cf. Herwig and Waszak, 2009), having more influence on response selection, our results show that also non-intended, task-irrelevant action effects can show an important influence in task performance (cf. also Pfister et al., 2010, 2011).

This association seems to occur very fast, as the switch costs were already smaller in the first two blocks for the experimental group compared to the control group, although participants had only little opportunity to experience the predictable task-response-effect mapping. There is also evidence in the study of Kiesel and Hoffmann (2004) that the mental association between the predictable mappings is built up quickly: after the first half of the experiment, they interchanged the task context-action effect assignment. Performance did not differ in the two halves of the experiment, demonstrating that participants learned the new assignment very fast, even though the old assignment could have interfered. If action effects play a role in task implementation – and our results argue for it – only few encounters with the predictable mapping might be enough to build up an association (cf. also Dutzi and Hommel, 2009, who also argue for a fast response-effect binding).

Taken together, our data support the hypothesis that action effects play an important role in task implementation. Further, we can conclude that the learned associations are task-specific, that means task-response-effect associations instead of stimulus-response-effect associations, because the switch costs are affected. In this regard, one might ask if action effects do not only influence task-set activation but are even a part of the task set as a distinct task-set component. With respect to our study, this assumption is not yet necessary, but this may be a theoretical option that should be investigated in future studies. Either way, one should not forget that in our daily life, performing tasks has mostly external effects. Hence, to really understand how humans are dealing with multiple tasks in their daily life, action effects should be considered as an important influence in task performance in future studies.

## Conflict of Interest Statement

The authors declare that the research was conducted in the absence of any commercial or financial relationships that could be construed as a potential conflict of interest.
